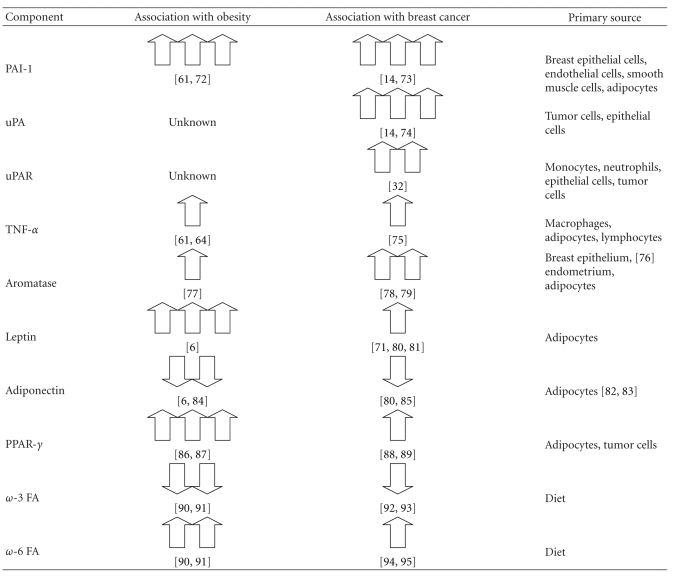# Obesity and Breast Cancer: The Roles of Peroxisome Proliferator-Activated Receptor-*γ* and Plasminogen Activator Inhibitor-1

**DOI:** 10.1155/2009/345320

**Published:** 2009-08-06

**Authors:** Jennifer C. Carter, Frank C. Church

**Affiliations:** ^1^Department of Pathology and Laboratory Medicine, School of Medicine, University of North Carolina, Chapel Hill, NC 27599-7035, USA; ^2^Department of Pharmacology, School of Medicine, University of North Carolina, Chapel Hill, NC 27599, USA; ^3^Division of Hematology-Oncology, Department of Medicine, School of Medicine, University of North Carolina, Chapel Hill, NC 27599, USA

## Abstract

Breast cancer is the most prominent cancer among females in the United States. There are a number of risk factors associated with development of breast cancer, including consumption of a high-fat diet and obesity. Plasminogen activator inhibitor-1 (PAI-1) is a cytokine upregulated in obesity whose expression is correlated with a poor prognosis in breast cancer. As a key mediator of adipogenesis and regulator of adipokine production, peroxisome proliferator-activated receptor-*γ* (PPAR-*γ*) is involved in PAI-1 expression from adipose tissue. We summarize the current knowledge linking PPAR-*γ* and PAI-1 expression to high-fat diet and obesity in the risk of breast cancer.

## 1. Introduction

### 1.1. Breast Cancer Epidemiology

Breast cancer is the most commonly diagnosed cancer in the female population and is second in cancer related deaths in the United States [1]. While the mortality has decreased slightly in recent years, the number of cases diagnosed annually has remained relatively steady. According to the American Cancer Society, over 178000 new cases are diagnosed each year, with an estimated 40 400 deaths from breast cancer in 2008 [1]. Five-year survival rates of breast cancer patients is almost 90%, although higher in patients over 40, as women diagnosed at a young age typically have a more aggressive cancer that is less responsive to treatment [1]. Though both incidence rates and mortality rates have decreased in recent years, the healthcare costs and the emotional costs of breast cancer remain high. 

A number of risk factors are associated with development of breast cancer. The greatest risk factors are age and gender, with females developing breast cancer 100 times more frequently than males [2]. As a woman ages, her risk of developing breast cancer increases, from 1 in 233 between the ages of 30–39 to 1 in 27 between the ages of 60–69. While age and gender are the greatest risk factors, there are also hormonal risk factors associated with breast cancer development, including age at first menarche, age at menopause, and lifetime exposure to estrogen [3, 4]. Furthermore, a family history of breast cancer and a history of previous benign breast disease are risk factors associated with breast cancer [5].

### 1.2. Breast Anatomy

The breast is a very heterogeneous tissue, composed of a number of different cell types. Epithelial cells make up the parenchyma of the tissue, forming the ducts and glands involved in milk production, storage, and secretion [5]. Surrounding these epithelial cells is a network of fibroblasts, which generate the proteins of the breast connective tissue [5]. Another key component of breast tissue is adipose, composed of mesenchymal precursor cells and the mature adipocytes [5]. In addition to energy stores, adipocytes synthesize and secrete a number of cytokines, which are involved in a number of pathogenic processes, including inflammation [6]. 

Our interest in the adipose tissue of the breast stems from the understanding that the tumor microenvironment provides a number of signals and resources to the tumor cells, promoting proliferation, survival, and motility. In that regard, adipocytes, or their precursor cells, may provide key factors in breast tissue needed for tumor development, progression, or even enable tumor cell invasion. Additionally, several recent studies suggest a woman with dense breast tissue is more at risk for developing breast cancer [7–9]. Collectively, these results imply that excess amounts of adipose in either the breast or other distant fat depots could provide a climate amenable to development of carcinoma of the breast. 

The overall goal of this paper is to present evidence supporting the link between how a high-fat diet and obesity increases the risk of breast cancer. We focus on the expression of the nuclear receptor peroxisome proliferator-activated receptor-*γ* (PPAR-*γ*) and the serine protease inhibitor (serpin) plasminogen activator inhibitor-1 (PAI-1).

## 2. Tumor Progression and Metastasis

In order for a cancer cell to progress to a disease state, the cell must be able to proliferate and generate a clonal population, resulting in a tumor [10]. To do the most harm, cancer cells must possess the ability to survive and migrate from their site of origin. Motility allows these cells to move from primary sites, such as the breast, to distant metastatic sites [11]. In terms of breast cancer, the most common sites of metastases are bone, brain, and lung [5]. In order to move to distant sites, these cells must degrade the surrounding extracellular matrix (ECM) and invade nearby blood and lymph vessels [12]. The plasminogen activator (PA) system allows tumor cells to activate plasminogen, resulting in the active proteolytic enzyme plasmin and ECM degradation [13]. In breast cancer, this system is often dysregulated, resulting in migration and invasion of tumor cells into the surrounding vasculature and lymphatics [14].

Recently the tumor microenvironment has come into the forefront as a possible source of either help or hindrance to the tumor. As mentioned previously, the breast is largely made up of fibroblasts, connective tissue, and adipose. PPAR-*γ* is a key regulator of adipogenesis [15–17], and there is growing evidence of its importance in many pathophysiological processes [17–20]. PPAR-*γ* has been shown to be dysregulated in the obese population [21], and since obesity is a known risk factor for breast cancer development, PPAR-*γ* activity may have a role in breast cancer inhibition. Several studies have suggested that activation of PPAR-*γ* inhibits cell proliferation and induces apoptosis in vitro [22–26]. PPAR-*γ* has been found to regulate PAI-1 expression in endothelial cells, smooth muscle cells, and pancreatic cell lines [27–29].

## 3. The Plasminogen Activator System in Breast Cancer

**Figure 1:**
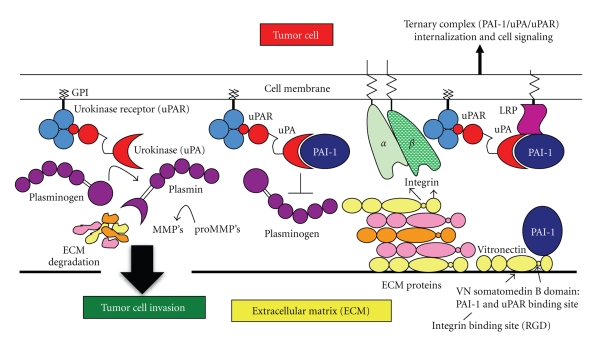
*Plasminogen Activator System at the Tumor Cell Surface*. Besides its traditional role as a protease inhibitor, the multiple roles of PAI-1 including cell de-adhesion, proliferation/apoptosis, and cell signaling suggest that PAI-1 expression in the tumor microenvironment enhances tumor cell progression. (*Left panel*) The catalytic activity of urokinase (uPA) is enhanced when bound to the cell surface by uPAR. uPA cleaves the zymogen plasminogen to its active form, the serine protease plasmin. Plasmin can subsequently activate matrix metalloproteases (MMP's) in the extracellular matrix (ECM) microenvironment. Thus, the uPA/uPAR complex and MMP activation contribute to tumor cell invasion and metastasis by degradation of ECM components. (*Middle panel*) PAI-1 directly inhibits the active site of uPA whether it is free or bound to uPAR, and reduces further activation of plasminogen to plasmin. The PAI-1 paradox exists because this inhibition reaction should reduce tumor cell progression and invasion. (*Right panel*) When uPA is neutralized by PAI-1, the trimeric PAI-1/uPA/uPAR complex is recognized by the lipoprotein related protein (LRP) and internalized. Furthermore, PAI-1 has vitronectin (VN) binding sites and causes tumor cell detachment away from the ECM. This figure is based on a schematic from [13].

The role of the PA system is to regulate fibrinolysis and to promote pericellular proteolysis [30]. Plasminogen is cleaved by a plasminogen activator to its active serine protease, plasmin. Tissue-type plasminogen activator (tPA) mediates plasminogen activation in the vasculature. Plasmin then hydrolyzes fibrin, restoring hemostasis. Outside of this fibrinolytic pathway, the PA system also plays a role in tumor cell invasion (Figure 1). In the pericellular environment, the serine protease urokinase plasminogen activator (uPA) is primarily responsible for the cleavage of plasminogen. Plasmin is then able to degrade the extracellular matrix (ECM) directly or indirectly by activation of promatrix metalloproteinases, which then degrade the ECM [31, 32]. Plasmin also activates more uPA, forming a positive feedback loop that further supports invasion-linked processes. In addition to these roles, the cell surface uPA receptor (uPAR) plays a role in integrin mediated cell motility. Bound to uPA, uPAR is able to bind various integrins, resulting in the rearrangement of the cytoskeleton, promoting cell motility. Elevated uPA is a poor prognostic indicator in a number of cancers, including carcinoma of the breast [33–36]. The serine protease inhibitor (serpin) PAI-1 binds to the active site of uPA, blocking the activation of plasminogen to plasmin. PAI-1 also affects cell adhesion and migration though binding of the uPA/uPAR complex. The PAI-1/uPA complex is recognized by lipoprotein-related protein (LRP), a scavenger receptor, and is rapidly internalized; uPA and PAI-1 are then degraded and uPAR is recycled to the cell surface [37]. Since elevated PAI-1 levels are an indicator of poor prognosis in breast cancer, this data would suggest increased amounts of PAI-1 result in a deattachment of the cell from the ECM, allowing for enhanced cell motility. These tumor cells could then invade into the surrounding blood vessels and lymphatics, becoming metastases of the primary cancer. 

### 3.1. Urokinase Plasminogen Activator

Urokinase plasminogen activator (uPA) is a 53 kDa serine protease. Initially a zymogen, pro-uPA is cleaved to the active form [38]. Though the physiological activator is unknown, in vitro a number of proteases can activate uPA. In addition to protease cleavage, binding the uPA cell surface receptor uPAR can activate pro-uPA. uPA functions to cleave plasminogen to its active protease plasmin. In 1978, Verloes et al. demonstrated inhibition of uPA resulted in tumor growth inhibition, implicating a pathological role for uPA [39]. Since this discovery, uPA has been shown to be involved in tissue remodeling, inflammation, fertilization, embryogenesis, and tumor invasion [40, 41].

### 3.2. Urokinase Plasminogen Activator Receptor

The cell-surface receptor for uPA (uPAR) is a 55–60 kDa protein. It has no transmembrane domain; it is anchored to the cell surface by a glycosyl phosphatidylinositol anchor. uPAR is required for the endocytosis of uPA/PAI-1 complexes and plays a key role in uPA activation. Research has also shown uPAR mediates cell proliferation through activation of ERK/MAP kinase pathways following binding of uPA [42]. Examination of uPAR protein levels in several breast cancer cell lines showed a correlation with invasiveness in vitro [43]*.* In breast cancer patients, combined overexpression of uPAR, PAI-1, and uPA was shown to correlate with decreased survival [44].

### 3.3. Plasminogen Activator Inhibitor Type-1

PAI-1 is a glycoprotein of approximately 50 kDa [45] and a member of the serine protease inhibitor (serpin) superfamily of proteins [46]. PAI-1 binds the active site of tPA [47, 48] and uPA, preventing cleavage of plasminogen. Binding of PAI-1 to vitronectin (VN), which stabilizes the protein in blood circulation [49]. While the physiological role of PAI-1 is to inhibit plasminogen activation, it is a poor prognostic indicator for a number of cancers, including breast cancer [14, 50, 51]. There is no single mechanism to explain why an elevation in PAI-1 protein results in decreased patient survival, but there are a number of studies that suggest alternative roles for PAI-1 outside of the traditional protease inhibitor role. Specifically, several studies indicate that PAI-1 promotes tumor growth through an inhibition of apoptosis [23, 26, 52]. PAI-1 has also been implicated in angiogenesis [53, 54], increased cell adhesion [55], and increased migration [56]. In addition to the role of PAI-1 in breast cancer migration and invasion, it has been implicated in an inflammatory response [57], neutrophil recruitment, and in proliferation of smooth muscle cells [58]. Furthermore, increased PAI-1 levels have been associated with obesity [59–62], with recent reports suggesting the elevation in PAI-1 levels is the result of PAI-1 production from adipocytes [63–65].

## 4. Obesity and Breast Cancer Risk

A number of factors are associated with an increased risk of developing breast cancer (Table 1). While age and gender are the two predominant risk factors, some risk factors remain modifiable, such as diet and obesity [66–68]. Adult weight gain is correlated with increased breast cancer risk and is a poor prognostic factor [69]. The mechanism behind the relationship of increased incidences of breast cancer in obese individuals is poorly understood; however, the literature concerning this association has increased in recent years [70, 71]. 

Besides their traditional role as energy stores, adipocytes are now considered to be an important “endocrine gland”, expressing numerous proteins involved in several physiological and pathological responses [64, 96]. Aromatase, the enzyme needed to activate estrogen, is one of the factors expressed by adipose tissue. Recently it was suggested that stromal cells in the adipose tissue, not adipocytes, express aromatase [77–79, 97]. Elevated aromatase in the breast correlates to elevated levels of estrogen in the breast [97]. It is hypothesized this is a key reason for the increased risk of developing breast cancer in obese postmenopausal women. 

Another factor expressed in the adipose tissue is the hormone leptin. In obese individuals, leptin is overexpressed [98]. In vitro, leptin has been shown to increase cell motility and decrease cell apoptosis in breast cancer cell lines [68, 82]. The mature adipocyte also expresses adiponectin. As opposed to the overexpression of leptin in obese individuals, adiponectin is downregulated [6, 99]. Grossman et al. showed the balance of adiponectin and leptin mediated breast cancer cell growth in vitro [80]. Studies have shown an antitumor effect of adiponectin in breast cancer. Treating cells with adiponectin decreases cell motility and induces apotosis [85]. Furthermore, adipocytes express several chemokines involved in the inflammatory response. 

A number of other adipokines are associated with cancer progression and metastasis, including PAI-1 [64, 65, 100]. As stated previously, obese individuals have elevated serum levels of PAI-1 [59, 101]. Interestingly, one study found an inverse relationship between adiponectin and PAI-1 expression in overweight and obese women [57]. With elevated plasma levels of PAI-1 from the adipose tissue, it is possible obese women are more prone to developing breast cancer and having a more aggressive disease. Prostate cancer cell growth in vitro is enhanced by this cancer cell-adipocyte communication; thus, it is interesting to speculate that breast cancer cell-adipocyte interactions would behave in a similar manner.

## 5. Peroxisome Proliferator-Activated Receptor-Gamma

The master regulator gene of adipogenesis is PPAR-*γ*, a member of the nuclear receptor superfamily [15, 102]. Mice null for PPAR-*γ* are embryonic lethal [103], suggesting PPAR-*γ* is essential for normal mouse development. PPAR-*γ* is a ligand-activated transcription factor, whereupon binding of the ligand, PPAR-*γ* translocates to the nucleus and heterodimerizes with RXR [104]. PPAR-*γ* binds to the target gene at a PPAR response element (PPRE), where it initiates transcription through the recruitment of transcriptional machinery [105]. Loss-of-function and gain-of-function mutations of PPAR-*γ* have been implicated in a number of disease processes, primarily type-2 diabetes mellitus, or insulin resistant diabetes [106]. The thiazolidinedione (TZD) family of drugs works to activate PPAR-*γ*, restoring insulin sensitivity to tissue, upregulating free fatty acid uptake by adipocytes, and altering expression of adipokines [107, 108]. PAI-1 expression is known to be regulated by PPAR-*γ*, though the literature is conflicting, suggesting PPAR-*γ* downregulates PAI-1 expression [109–111], while others suggests PAI-1 is upregulated by PPAR-*γ* agonists [28, 29, 112]. As adipogenesis is regulated by PPAR-*γ*, it has been postulated that obesity and the associated adipocyte pathology is due to a downregulation of PPAR-*γ* activity, either through mutation, phosphorylation, or methylation [113–115]. While PPAR-*γ* is required for adipocyte differentiation, under normal conditions, PPAR-*γ* serves to regulate cell size and transcription of adipocyte specific genes [16, 116]. One hallmark of obesity is elevated levels of inflammatory cytokines. In addition to positively regulating gene transcription, PPAR-*γ* has been shown to inhibit gene transcription as well [117, 118]. By blocking gene transcription machinery from binding the promoter site, PPAR-*γ* negatively regulates several genes, including NF*κ*B, a key transcriptional factor involved in numerous disease processes, including inflammation [117]. 

The ability of PPAR-*γ* to inhibit NF-*κ*B expression is important in breast cancer progression, since NF-*κ*B has been shown to increase tumor cell invasiveness as a result of increased uPA expression [119]. Altered expression of nuclear NF-*κ*B has also been shown to prevent apoptosis [120]. Other studies have shown NF-*κ*B is involved in mammary epithelial proliferation [121, 122], and also chemoresistance in MCF-7 breast cancer cells [121]. In addition to its role in inhibiting NF-*κ*B expression, PPAR-*γ* activation has also been shown to downregulate transcription of the insulin receptor (IR) by physically interacting with the transcription factors Sp1, C/EBP*β*, and AP1 in vitro, preventing IR transcription [118]. Insulin receptor signaling has been implicated in a number of neoplastic processes including proliferation, invasion, and cell survival [123]. Recently, elevated levels of insulin in newly diagnosed breast cancer patients were shown to be related to an underlying insulin resistance [124]. These data, and the fact that insulin resistance is associated with increased risk of breast cancer [125] and poor patient prognosis [126], suggest a possible role for PPAR-*γ* activators in either prevention or treatment of breast cancer patients. 

Since PPAR-*γ* regulates adipocyte differentiation and normal function, PPAR-*γ* malfunction may play a role in tumor development. Several studies have shown PPAR-*γ* is expressed in a variety of tumor types, including pituitary tumors [127], ovaries [128], prostate [22, 88], colon [129], and breast [88]. While the in vivo role of PPAR-*γ* in these tumor cells is unknown, in vitro data suggests PPAR-*γ* activation can induce apoptosis [26] and promote terminal differentiation of breast tumor cells [88]. Nunez et al. reported induction of apoptosis in the MDA-MB-231 breast cancer cell line, but not in normal fibroblasts, treated with ciglitazone following amino acid deprivation [23]. More recently, Bonofiglio et al. showed rosiglitazone enhances FasL expression in a PPAR-*γ* dependent manner, resulting in induction of apoptosis in a number of human breast cancer cell lines [130]. 

The findings above supported a clinical trial testing troglitazone as a new chemotherapeutic agent in breast cancer, which was terminated when troglitazone was taken off the market for severe liver toxicity [131]. More recent clinical trials have looked into the chemotherapeutic effects of two commercially available PPAR-*γ* agonists. A Phase-I trial investigating rosiglitazone treatment in conjunction with bexarotene found the maximum tolerated dose in breast cancer patients with refractory disease [132]. A Phase-II trial treating patients with high-grade gliomas with combination therapy of pioglitazone and the COX-2 inhibitor rofecoxib showed moderate activity, which was tolerated well by the patients [133]. While these clinical trials showed no overall improvement, there is evidence to suggest treatment of diabetic patients with PPAR-*γ* agonists may have some preventive effects on cancer development. One study looked at male type 2 diabetes patients and found patients treated with TZDs had lower incidences of cancer, specifically lung cancer [134]. As this study was designed using male patients, there is no breast cancer data. A metaanalysis of clinical trial data found patients treated with the TZD rosiglitazone had lower incidences of malignancy than control non-TZD treated patients [135]. As the number of patients with type 2 diabetes increases, it will be important to closely monitor the patients treated with TZDs in terms of incidences of malignancy.

## 6. Diet, PPAR-*γ*, and Cancer Development

Recent epidemiologic studies suggest diet may also be associated with developing certain cancers. In 1997, Huang et al. showed a positive correspondence to adult weight gain and postmenopausal development of breast cancer [136]. These results and others suggest obesity is a modifiable risk factor for breast cancer development. More recently, a study showed body mass index (BMI) to be correlated with several sex hormones, helping to explain the positive relationship between obesity and breast cancer risk [137]. Adult weight gain has also been associated with an increased risk of breast cancer, particularly in women not on hormone replacement therapy [138–140]. 

In addition to reports of obesity and cancer development, a number of studies suggest diet is a key player in cancer progression. Increased fat intake is a risk factor for a number of cancers, including breast, prostate, and colon. While the mechanism is not fully understood, dietary fats have been implicated in tumor progression. Dietary fats, specifically polyunsaturated fatty acids (PUFAs), are certainly involved in the inflammatory process [141, 142], which is linked to cancer cell motility and survival. PUFAs include the omega-3 (*ω*-3) and omega-6 (*ω*-6) classes of dietary fatty acids, both of which play essential roles in normal physiology. Interestingly, both *ω*-3 and *ω*-6 fatty acids have been shown to bind and activate PPAR-*γ* [25, 143–146]. Consumption of *ω*-3 fatty acids, specifically, eicosapentanoic acid (EPA) and docosahexanoic acid (DHA), decrease the risk of coronary artery disease, stroke, and other diseases associated with an inflammatory response, such as Crohn's disease [90, 147, 148]. More recently, *ω*-3 fatty acids have been shown to inhibit tumor growth and decrease tumor cell motility [149–151]. While *ω*-3 fatty acids are often associated with health benefits, *ω*-6 fatty acids have been implicated in a number of disease processes. The *ω*-6 PUFA arachidonic acid (AA) has been shown to increase cell motility and survival through inactivation of the tumor suppressor PTEN [152], while another group has shown that AA directly activates PI3K and upregulates numerous inflammatory genes [153]. Because AA is an essential fatty acid, it is required for normal cell homeostasis, suggesting some AA is critical, but excess AA can be problematic. 

Several studies suggest the *ω*-3 PUFA to *ω*-6 PUFA ratio is what drives cancer cell biology. In prostate cancer, cells with a lower *ω*-3 to *ω*-6 ratio had increased cell survival and motility [154]. It is important to note diets in the United States typically consist of elevated levels *ω*-6, compared to a Mediterranean or Asian diet [91, 155]. Numerous studies have investigated PPAR-*γ* activation by dietary fatty acids. AA has been implicated as a regulator of PPAR-*γ* activity as well, downregulating transcription of a PPAR-*γ* target gene GLUT4 [146]. Another *ω*-6 fatty acid gamma-linolenic acid (GLA) has been shown to activate PPAR-*γ* in breast cancer cells, resulting in cytotoxicity and adhesion [156]. One group showed a differential effect of *ω*-3 versus *ω*-6 fatty acids on PPAR-*γ* transcriptional activity [157], with *ω*-3 fatty acids downregulating PPAR-*γ* activity in the MCF-7 breast cancer cell line. It should be noted, numerous studies suggest *ω*-3 fatty acids act as PPAR-*γ* agonists. Sun et al*.* showed *ω*-3 fatty acids increase syndecan-1 production in breast cancer cells through PPAR-*γ* activation [151]. Another study showed eicosapentaenoic acid (EPA) activates PPAR-*γ*, resulting in inhibition of interleukin-6 expression in glioma cells [158, 159]. Using human colon cancer cell, Allred, et al. showed EPA suppressed cell growth through PPAR-*γ* activation [159]. 

Increased long-chain *ω*-6 fatty acids in adipose tissue of the breast have been shown to correlate with development of breast cancer [94, 95]. Tumors in mice fed increased *ω*-3 fatty acids had increased apoptosis and decreased proliferation, suggesting a protective role of *ω*-3 fatty acids in tumor progression [154]. In breast cancer cells, gene expression is differentially regulated by *ω*-3 and *ω*-6 fatty acids [160]. A number of animal studies also suggest a protective role for *ω*-3 fatty acids in breast cancer progression [161]. Recently, a study showed *ω*-3 fatty acids inhibits HER-2/neu-induced breast cancer in transgenic mice, independent of PPAR-*γ* activity [162]. Horia and Watkins [163] reported that MDA-MB-231 cells treated with docosahexanoic acid (DHA) and genistein were less invasive, had reduced COX-2 and NF-*κ*B expression and increased PPAR-*γ* expression. Furthermore, in vivo*,* increasing *ω*-6 fatty acid in mice with prostate cancer xenografts results in increased tumor growth and final tumor volumes [154]. Taken together, these studies begin to explain the role of diet and obesity in breast cancer risk and development, potentially mediated through PPAR-*γ* activity. 

The importance of the tumor microenvironment must not be overlooked in cancer research. In recent years, evidence has increasingly shown the relationship between tumor cells and the surrounding stromal cells. In prostate cancer, there are reports of cross-talk between bone and metastasized prostate carcinoma cells, promoting growth and survival of metastatic lesions [164]. Adipocytes have been shown to promote tumor growth by secretion and processing of collagen IV, which activates AKT signaling pathway in breast epithelial cells [165]. Using proteomic analysis of adipocyte cells and interstitial fluid of fat tissue from the breast, another study identified proteins involved in metabolism, apoptosis, and immune response [166]. These studies suggest a link between adipocytes in the breast tissue and support of tumor development and growth. 

In terms of the role of PPAR-*γ* in cancer-stromal cell interactions, the literature is both sparse and contradictory. One in vitro study shows inhibition of adhesion between multiple myeloma and bone marrow stromal cells, through reduced activity of both NF-*κ*B and C/EBPb by PPAR-*γ* agonists [167], reducing growth and metastasis of multiple myeloma. Another in vitro study showed stromal cell expression of prostaglandin D synthase derived products suppressed prostate tumor growth and that this was mediated through PPAR-*γ* activation in the tumor cells [168]. Most in vivo characterization of PPAR-*γ* in tumors has been done through immunohistochemical analysis, which does not show activity of PPAR-*γ*, merely expression. There are a number of citations showing PPAR-*γ* protein expression in tumor samples [88, 127, 129, 169–171], though there is no definitive explanation for its presence. One study showed expression of PPAR-*γ* in pancreatic cancer is correlated with shorter patient survival [171], while expression of PPAR-*γ* in colon adenocarcinoma samples corresponded to increased expression of cell-cycle molecules [129]. These results suggest PPAR-*γ* activation may be used to induce expression of cell-cycle machinery. Another study found PPAR-*γ* to be highly expressed in both primary and metastatic breast cancer tissue samples [88]. A study in human mammary ductal carcinoma in situ (DCIS) found elevated expression of nuclear PPAR-*γ* was inversely related to disease recurrence following breast conservation therapy [172]. Suzuki et al*.* show PPAR-*γ* immunoreactivity in breast carcinoma tissue was associated with improved clinical outcome [173]. While these studies reveal the presence of PPAR-*γ* in tumor cells, without evidence suggesting PPAR-*γ* activity, one cannot fully understand the role of PPAR-*γ* in tumor cell biology in vivo.

## 7. Summary

**Figure 2:**
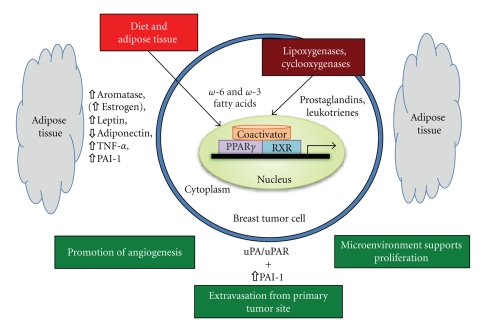
*Potential Role of PPAR-γ, Fatty Acid Ligands, Adipose Tissue, and the Plasminogen Activator System in Breast Cancer*.

While there is a substantial amount of data on PPAR-*γ* pertaining to its role in normal cell function and diabetes, there is no solid understanding of its function in cancer cell lines or tumor samples. The in vitro data supports a role for PPAR-*γ* in differentiation of tumor cells [88] as well as induction of apoptosis [174–176], though there is no strong in vivo data to support the in vitro results. What is known is that obesity exhibits a number of hallmarks for altered PPAR-*γ* function, including dysregulation of adipokine secretion. In this review, we have presented support for our hypothesized mechanism of increased breast cancer risk in obese individuals. With elevated levels of PAI-1 in obese women, the potential is there for increased proliferation, decreased apoptosis, and increased cellular migration, all contributing to tumor development and metastasis in the breast (Figure 2). The close proximity to a large pool of adipose tissue in the microenvironment could predispose obese women to developing breast cancer. 

The potential to use PPAR-*γ* agonists as chemotherapeutic agents in breast cancer is a very viable option. It is possible that inducing PPAR-*γ* activity systemically in the obese individual could alter PAI-1 expression, resulting in a less pathogenic phenotype in the breast tissue. Additionally, by activating PPAR-*γ*, NF-*κ*B has been shown to be downregulated, resulting in reduced uPA expression. Inhibiting uPA expression also has the potential to alter the breast tissue microenvironment, preventing possible tumor cells from invading into the surrounding vasculature. Less toxic PPAR-*γ* agonists, such as pioglitazone or rosiglitazone, both FDA approved and commercially available to treatment of diabetes, may prove to be useful chemotherapeutic agents for breast cancer patients.

## Figures and Tables

**Table 1:** Relationship between obesity, PPAR-*γ*, fatty acids, and increased risk of breast cancer.